# Supplementary figures and images for: Classifying sex and strain from mouse ultrasonic vocalizations using deep learning
Source: PLoS Comput Biol. 2020 Jun 22;16(6):e1007918. doi: 10.1371/journal.pcbi.1007918 (PMC7347231; doi:10.1371/journal.pcbi.1007918)

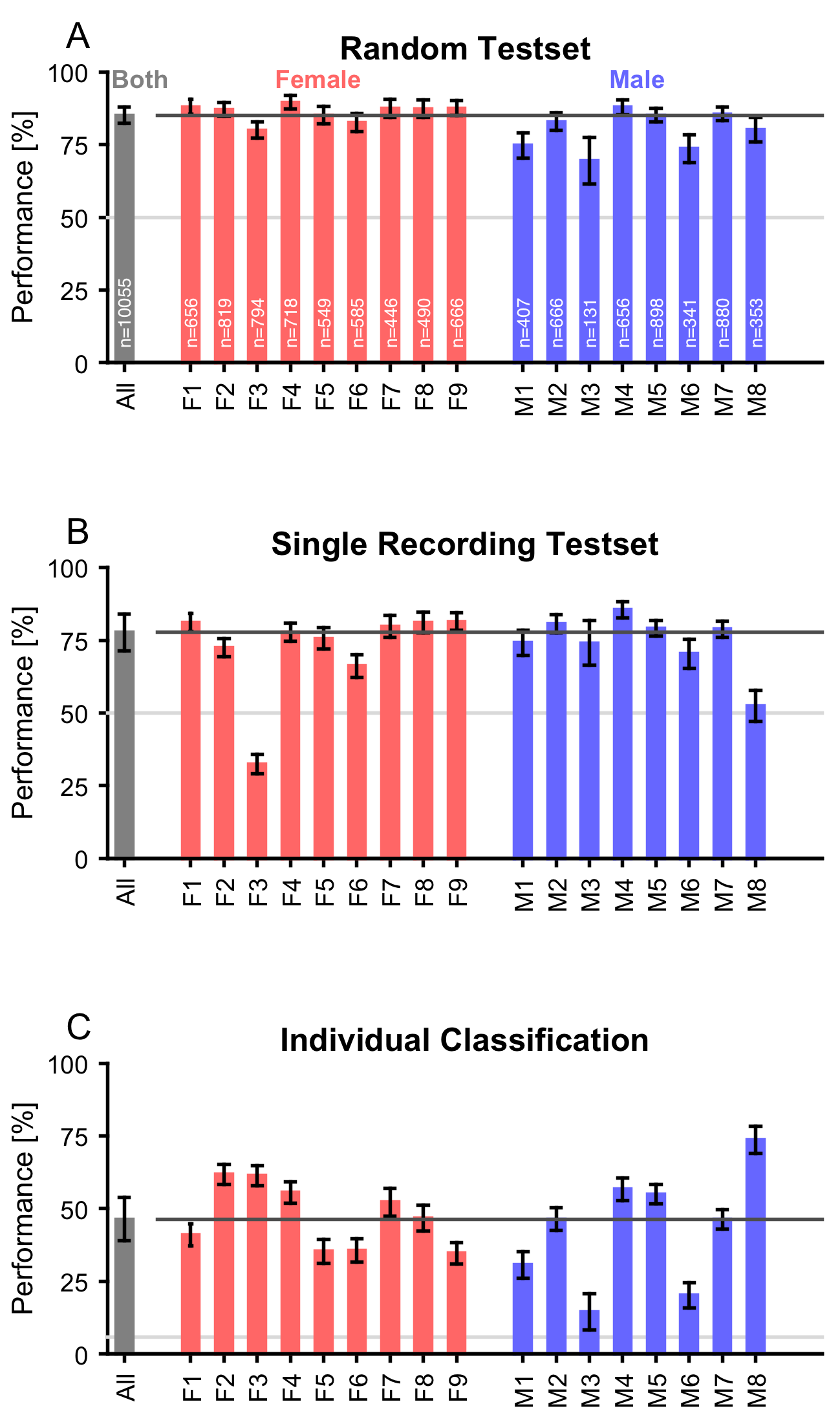

Supplement: S1 Fig — A Comparing the performance of the network across individual indicates that the sex-classification is better than chance in 15/17 animals. The light gray line indicates chance, and the dark gray line average performance. B If individual properties are included in the classification (through the use of random testset during crossvalidation, 10x), overall performance increases to 85.1% (median across animals). C We trained another DNN to classify mouse identity, achieving above chance performance for almost all mice, indicating directly that differences between individual mice can also contribute to the classification of sex. For classification of individuals, the chance level is at 100/Nmice%. (TIF) [file pcbi.1007918.s001.tif]

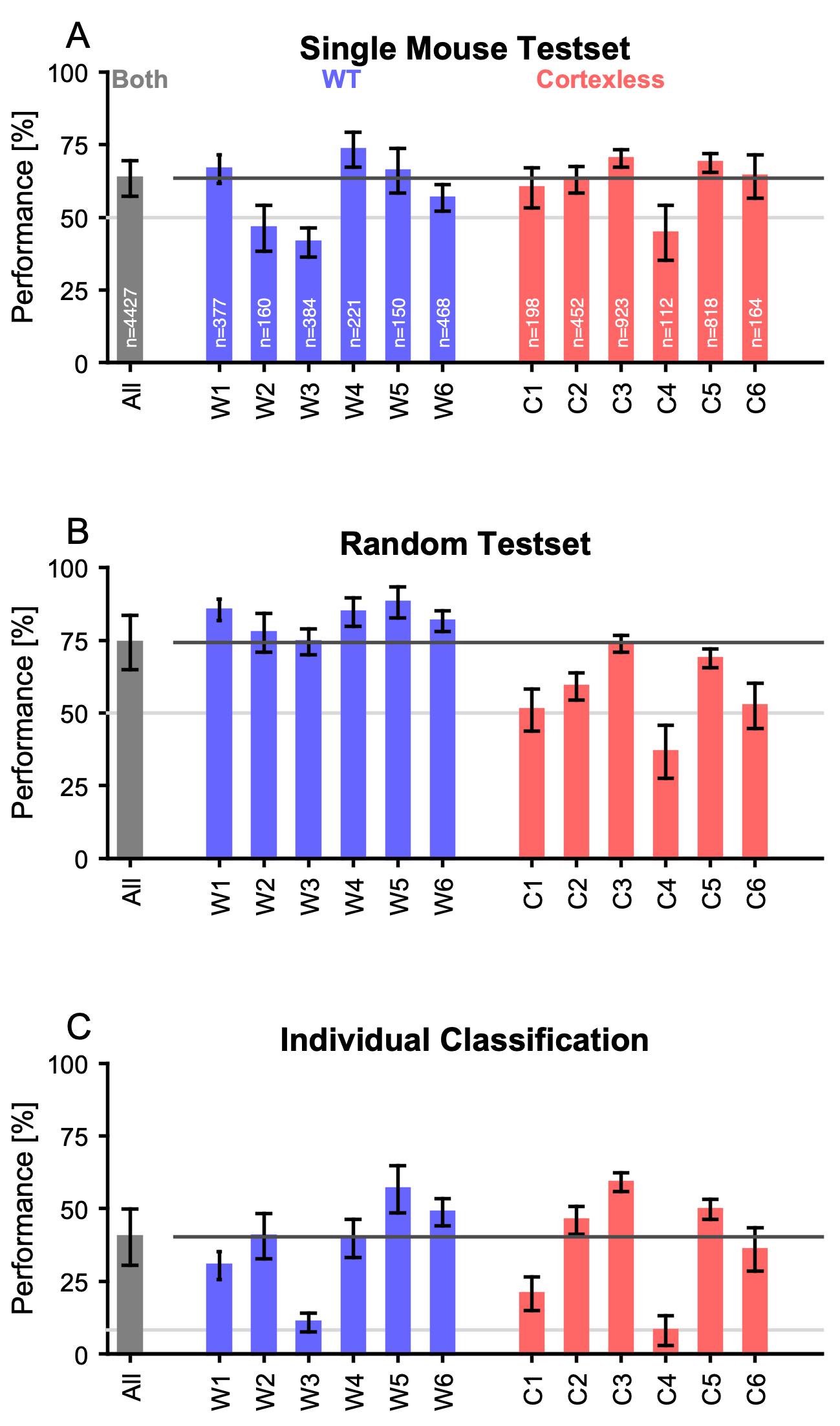

Supplement: S2 Fig — 1 for the classification of wild-type vs. cortexless animals. (TIF) [file pcbi.1007918.s002.tif]
