# Supplementary material for: Classifying sex and strain from mouse ultrasonic vocalizations using deep learning
Source: PLoS Comput Biol. 2020 Jun 22;16(6):e1007918. doi: 10.1371/journal.pcbi.1007918 (PMC7347231; doi:10.1371/journal.pcbi.1007918)
Supplement: Attachment — Submitted filename: ResponseLetter_USVClassify.docx [file pcbi.1007918.s004.docx]

**Dear Alexander and Bernhard,**

**Both I and the reviewers found that your use of the DNN for classifying SUV is interesting - although I still find it somewhat incremental. Both reviewers have addressed minor points that you should address.**

**I am very much in agreement with reviewer 1 that the only cross-validation test that is correct is what you call the single recording test set. Your paper should not use any other tests - period! This is true not only for the DNN but with your comparisons with other methods like linear regression. Otherwise you are admitting that you have a "random-effect" the identity of each mouse but ignoring it. It is as if you knew that you need to do mixed-effect modeling but refrain from doing so. In terms of the analysis of vocalizations, this point was very explicitly made by Mundry and Sommer (Anim Behavior 2007) as applied to linear classifiers - they call their approach permuted DFA. Note also that this is the type of cross-validation that my lab has always performed both with Hyenas and with Zebra Finches.**

R: While we tried to separate the corresponding prediction accuracies clearly in the text, we agree that it makes sense to focus the presentation of the paper on the accuracy that has no access to individual traits. On the other hand, we think that the contribution of individual features is still interesting, and - after brief coordination with the Dr. Theunissen - we opted for presenting these now in the two supplementary figures and reference their results only briefly in the appropriate places in the main text.

**I have also not looked at what is usually done for mouse for USV but the 9 bio acoustical features you choose are quite simple - I would like you try a much more complete set of features that include a better description of the spectral envelope (spectral mean, quartiles, etc), temporal envelope and fundamental frequencies (we have used such features in our work but they are quite commonly used by bioacousticians). For the fundamental frequency, you might even to PC of the time-varying fundamental (eg. Dahl A, Sherlock BR, Campos JJ, Theunissen FE, 2014)**

R: We agree that the choice of features could be expanded, which could provide a better insight into the most salient features. We have followed the editor's suggestion and expanded the set by the following features (in line with the editor's proposals):

- Temporal Envelope: This is the sequence of amplitudes over time, over the length of the vocalization, followed by zeros to have a uniform length of 100.
- Spectral Envelope/Marginal: This is the marginal spectrum of the USV over the entire frequency range (dimension is 233).
- Spectral Line: time-varying fundamental frequency without amplitudes, i.e. sequence of frequencies of the duration of the vocalization up to 100 time steps (ms).
- To emphasize, plus the nine features that we used before.

The results for this analysis are shown in the extended figure 4, panels J-L . The network was restructured to allow a combination of separate convolutional layers for each of the three 1D features, and non-convolutional for the 9 discrete features, and of course completely retrained. We originally planned to use more extracted features to replace the composite 1D features above, but since the performance of this network did not reach the performance of the full 2D evaluation, we did expect this approach to exceed the 2D classification, as it would have fallen somewhere between the 9 features and the extended features approach, but likely not exceeded the performance of the latter.

**Reviewer #1: Review: PCOMPBIOL-D-19-01759**

**Ivanenko et. Al., have developed several DNNs (Deep Neural Networks) capable of classifying individual USVs into discrete categories with accuracy not previously achievable. Most notably they are capable of classifying the sex of the emitting mouse ~78% of the time. They were also able to extend this technique to classify USVs from a transgenic mouse, as well as classifying USVs by sever experimenter defined acoustic properties. The work reported here is extremely thorough and described in excellent detail, and the underlying method could prove extremely useful for researchers hoping to analyze vocalizations during opposite sex interactions. Computational methods of identification have significant benefits over competing methods, such as multi-microphone arrays, namely zero cost and ease of implementation. Although Dr. Englitz appears to be working on that solution in parallel, as well as considering combining the methods in the future. The technique also has some drawbacks. This is only useful for studying opposite-sex interactions, and 78% accuracy may be too low to effectively study syllable ordering, or syntax. The precise pattern of vocalizations is thought to be an important feature of social vocalization, and miss-categorizing 22% of syllables could drastically change results.**

R: We agree that there is definitely room for improvement, and that this room would be essential for making this a single-shot solution for assignment of vocalizations during social interaction. We, however, think that likely these will require a combined approach in any case: preliminary data from our recordings with a many-microphone array indicates that while the spatial accuracy is increased to about 7mm (median average error, in preparation), this is still insufficient for reliably classifying snout-snout interactions, and it would be these moments where we think the DNN analysis could increase the accuracy further.

**I have no major concerns**

**Minor concerns and suggestions**

**Calling the network 84% accurate is a bit misleading. DNNs are really meant to classify new information, and the network is only 78% accurate when tested against a novel mouse. Although the authors admit this, they lean heavily on the 84% number throughout the manuscripts.**

R: While we had tried to clearly state what each of the accuracies apply to, we agree that this is misleading with respect to sexual classification, which is the main topic of the manuscript. We have therefore followed your suggestions and changed the text accordingly. For details, see the response to the editor above.

**The title feels a little broad, and perhaps should include classification of sex or genotype.**

R: Agreed, changed.

**This manuscript seems to have two main threads. The first is proof that information about the sex of emitter mice is contained within individual USVs. I believe the authors do an excellent job proving this point, and they show clearly that the sex information is embedded in a complex combination of many features, including individual variation in USV production. While this work is thorough and well done, my enthusiasm for it is not particularly high. The features underlying classification are still unknown and I’m not exactly sure why it would be useful for mice identify sex based on a single call. It feels like this thread occupies the bulk of the text and sort of occludes the authors more important contribution.**

R: We hope that the changes that we made throughout the manuscript in response to your first comment have also reduced the emphasis on this point. We have in particular emphasized now that the use of individual properties of the mice may only be useful under specific circumstances (e.g. if there is training data for the isolated individuals available), but otherwise emphasize the single-animal accuracy.

**Namely, the authors have laid the groundwork for a universal sex classifier for mouse USVs. The current network is ~78% accurate within novel animals of the same strain, a major improvement to all existing methods I am aware of. The great thing about neural networks is they aren’t fixed or permanent. They can be shared and retrained by researchers around the world who have already collected datasets from separate strains or transgenic animals. Unfortunately, I couldn’t find the final networks among the available data. The Authors seem to have made all of the code and raw files available, so it should be possible to re-train the networks from scratch, but that seems like an unnecessary deterrent to wider adoption/refining of the technique.**

R: We would like to apologize for this omission, which was a mistake that happened in the final step of uploading. We have now uploaded the network and all associated code for running it to the updated repository (new URL, provided to the Editor) and would invite the reviewer to test-drive it and would be happy to implement suggestions for better reusability.

**Typos**

**79: feature combination s (I’m not sure about this)**

R: Thanks for noticing this. We fixed it in the revision.

**98: Sentence cuts off**

R: Thanks for noticing this. We fixed it in the revision.

**905: vocalization..**

R: Thanks for noticing this. We fixed it in the revision.

**Reviewer #2: USV – plos computational biology**

**The authors propose an interesting approach to sex discrimination in USV. They rightly identify that this a very challenging line of investigation.**

R: Thanks for your appreciation of the present study.

**Automated methods were used to automatically extracted USVs - please provide validation data on these methods as automated extraction is not a straightforward process and rarely as accurate as claimed.**

R: We agree that this process is far from trivial. We now provide a validation data set, where we compare hand-labelled vocalizations with the automated extractions both w.r.t. length, frequency range and recognition rate (where the hand-labelled ones are taken as the ground truth).

**In presenting the final outcomes, it would be helpful to report the metrics resulting from the rerun analysis as this more likely represents what would be observed in a novel cohort. Ie. performance is reduced.**

R: We interpret this question to refer to the use of the single animal test sets, rather than the random selection test sets (confirmed with the editor). In line with the editor's own suggestions, we have performed a substantial restructuring of the manuscript to focus on the single animal test sets and moved the random test set results to the supplementary figures (Fig. S1/2) and only briefly referring to them in the main text for completeness.

**Re-analyzing previously published data which reported no differences between groups, and then reporting different outcomes, is an important process.**

R: Agreed.

**Wording of this phrase needs revising “As intruders we used anaesthetized females to ensure that only the resident mouse could emit calls.”**

R: We tried to improve the sentence to the following, although we did not fully understand why it required revising (but we are also not native speakers): "Anesthetized females were used as 'intruders' to ensure that only the resident mouse was awake and could emit calls." Please let us know if this is better.

**More information on the WT/Cortexless paradigm is needed in the main document rather than making the reader find it in the supplementary materials.**

R: We were not sure how much we should bring it into the foreground, but we have now emphasized it more in the main text and methods.

**The authors mention that data were derived from a subset of both cohorts. How were these subsets selected? Were only the ‘best ‘recordings selected, was there any bias in this process or were they randomly selected?**

R: For the Cortexless data no selection was performed as this was all the data that was available from Dr. Hammerschmidt. For the Male/Female data the selection was performed randomly by Dr. Hammerschmidt, but it was not based on any quality selection.

**The number of calls appears sufficient but is 17 representative of mice in general?**

R: We partially agree that a larger dataset would have been preferable, however, unfortunately no additional recordings with a sufficient number of USVs per animal were available from our experimental collaborator (K. Hammerschmidt), and the experiments are not currently performed. Generally, however, the performance of the network should only increase with more datasets, since the error due to oversampling should be reduced (i.e. overlearning to the training set should become less with more data in relation to the training set size). We are therefore confident that the current results are quantitatively a lower bound to the actual performance of this approach.

**DNN are a great way forward in this field, but as in other fields, understanding the machinations within the convolution and connected layers and how the output represents a concrete concept is challenging.**

R: Thanks for this positive perspective. We agree that there are still important steps to be taken to interpret the analysis, while we hope that our preliminary steps (and the results to the extended set of acoustic features analysis (suggested by the editor)) provide some insight in this direction.
