# Supplementary material for: Classifying sex and strain from mouse ultrasonic vocalizations using deep learning
Source: PLoS Comput Biol. 2020 Jun 22;16(6):e1007918. doi: 10.1371/journal.pcbi.1007918 (PMC7347231; doi:10.1371/journal.pcbi.1007918)
Supplement: Attachment — Submitted filename: ResponseLetter2_USVClassify.docx [file pcbi.1007918.s005.docx]

Dear Frederic,

thanks for additional suggestions. We have made all the corresponding changes and rerun the feature-network, updating the associated text and figures. To our disappointment, these additional, in principle very useful additions did not increase the performance of the network to reach the performance of the full DNN on the spectrograms. It appears that despite your and our efforts to trace the source of the predictability through the use of these features, there is something we are still missing.

The new repository Code/Data link is:

[**https://data.donders.ru.nl/login/reviewer-32867936/3BVcXnd3ZznQw5Wq-frJgS_fKzamnDqAlIzugXpLfGs**](https://data.donders.ru.nl/login/reviewer-32867936/3BVcXnd3ZznQw5Wq-frJgS_fKzamnDqAlIzugXpLfGs)

**1. Methods: “Spectrograms were converted to a sparse representation by setting all values below 70th percentile to zero, which eliminated most close to silent background noise bins”. It is common to talk about thresholds in dB. So I suggest stating the corresponding dB threshold (e.g. This floor corresponds to a xx dB thresholds below peak).**

R: Changed, computed as 20 * log10(Pmax/Pthresh), and the difference was 55±5.2 dB.

**2. “Hence, vocalizations longer than 100 ms (11.7%) were included shortened.” Reword: “Hence, vocalizations longer than 100 ms () were truncated to 100…”. (I assume the end was deleted – otherwise use the correct words to explain how it was shortened).**

R: Changed. Yes, they were truncated.

**3. I know that you already provided more details on the NN but I am here also asking you to provide more details for the calculation of the auditory features. You should also use descriptions (and terms) that are well understood among bioacousticians.**

R: Thanks for pointing this out. We now use common terms and provide their definitions.

**I am assuming that [0,1] means any value between 0 and 1?**

R: Yes, this means that the possible range of the value was between 0 and 1, i.e. not just the integers 0 and 1.

**You figure 1 seems to say that these are attributed by visual inspection (by hand) but only 1, 0.5 and 0???:**

R: Exactly, these values were scored by two humans on all 10000 vocalizations (Methods: "The first 6 properties were scored by the human evaluators"). The values shown in figure one are just examples of what humans chose, and we chose these three steps to show the range. The actual values choses by humans covered the range from 0-1, typically in steps of 0.1 (probably due to internal rounding in the humans' minds).

**a. E.g. how is the broadband defined? And how do you go from 0 to 1? Why not use Weiner Entropy which might be more common in sound analyses?**

**b. Similarly for complexity? Is this the Weiner entropy?**

**c. Similarly for tremolo? Specific the equation that you used.**

R: We have substantially expanded the total number of extracted properties (from 9 to 24), and provide their definitions in the Methods. For most properties scored by humans, we now have an automatic estimate (only exceptions: peaks and breaks, which we considered to be reliably assessable by humans). We provide both the human version (for which we naturally do not have a definition) and the automatically estimated version of the overlapping parameters to the classifiers, based on the assumption that they can use information from one or the other depending on their predictability.

Regarding your specific questions above, we have added the Wiener Entropy, a well-defined version of FM-vibrato, spectral purity for the notion of broadband (in addition the frequency bandwidth was added).

**d. What is the average Frequency of a vocalization? The spectral mean? Or the average fundamental? These are very different. You should probably have both. You do talk about the mean spectral energy for detecting vocalizations. If that is what you use, use the same term.**

R: Most properties are now computed based on the fundamental frequency line (FF(t)), see Methods. For the average frequency we included also the weighted average of the spectrogram, to have both the fundamental only, and any potential other spectral components. Note, however, that the latter measure will tend to be more noisy, since non-USV parts of the spectrogram are also included, and USVs typically only have a single, dominant frequency per time-bin.

**e. Spectral mean requires a distribution. How is this one estimated?**

R: see above, the distribution is given by either the distribution of the intensities at the frequency locations in FF(t), or for the second measure, simply as the mean of the spectrogram over time.

**f. Also Power requires a frequency range – just all energy above 25 kHz. Normalized by duration? dB is a unitless measure (relative) so dB2 is meaningless. Both amplitude and power can be expressed in dB.**

R: Good point! Finding appropriate units for this quantity was indeed a bit unclear to us. We have now replaced this by spectral energy, given by the average over time of FE (the energy in the spectral bin of the spectrogram at each point of FF). We think this is the cleanest approach, since it focuses the estimate on the USV, rather than surrounding noises.

**4. I don’t think that any of the measures should be assigned by hand. Ok for Direction, Peaks and Breaks but Broadband and Complexity should be quantified. Why not use measures such as spectral bandwidth and Weiner entropy? You could also do something like pitch saliency.**

R: Good idea, we implemented all of the above, see Methods and response points above.

**5. I still find your choice of acoustic features somewhat limited. I think that some of the most experienced bioacousticians might wince and I would not like that given that my name will also be on your report as the associate editor.**

R: We understand, in total we have included 15 new properties, bringing the total to 24, see the bulleted list in Methods for a complete list. Sadly, this all did not help improve the predictions ... to our surprise as well! We worked meticulously to optimize the extraction of *FF(t)* (see examples below) and all subsequent parameters, and verified this for a lot of USVs manually... but there still seems to be something that the full DNN picks up, which we are not capturing with the plethora of properties.

**I would also add fundamental. You could do the mean but also do a time-varying fundamental (that you can express with PC for dimensionality reduction if you want).**

R: For the case of mouse vocalizations, there is virtually never a case with overtones (at least ones that are within the SR of the acquisition (due to the very high fundamental frequency of typically >50kHz), hence, the fundamental and the spectral line are identical. We have already integrated the spectral line, which is hence the same as including the fundamental. We changed the name now to Fundamental Frequency line (FF(t)), see Methods. Below we provide a few examples of he estimation quality for our spectral lines (red = estimate, gray = intensity in the spectrogram, after filtering)

**My lab as a Python toolbox that you can find in github.com: /theunissenlab/BioSoundTutorial that you might find useful to efficiently extract features. But don’t take my word for it – you can also see what has been published by other groups. One issue is that mice USV are late in the game and that most folks that have analyzed them are not bioacousticians so you will need to get inspired by analyses in other animals.**

R: Thanks for suggesting this toolset. We found it easier to implement the definitions in our toolset, taking inspiration from the definitions in your toolbox.
